# Anthropometric Criteria for Identifying Infants Under 6 Months of Age at Risk of Morbidity and Mortality: A Systematic Review

**DOI:** 10.1177/11795565211049904

**Published:** 2021-10-21

**Authors:** Christoph Hoehn, Natasha Lelijveld, Martha Mwangome, James A Berkley, Marie McGrath, Marko Kerac

**Affiliations:** 1 London School of Hygiene and Tropical Medicine, London, UK; 2 Emergency Nutrition Network, Oxford, UK; 3 Kenya Medical Research Institute/Wellcome Trust Research Program, Kilifi, Kenya; 4 The Childhood Acute Illness & Nutrition Network, Nairobi, Kenya; 5 Centre for Tropical Medicine & Global Health, University of Oxford, Oxford, UK; 6 Centre for Maternal, Adolescent & Reproductive Child Health, London School of Hygiene & Tropical Medicine, London, UK

**Keywords:** Wasting, malnutrition, anthropometry, infants, management of at-risk mothers and infants (MAMI), diagnosis

## Abstract

**Background:**

There is increasing global focus on small and nutritionally at-risk infants aged <6 months (<6 m). Current WHO guidelines recommend weight-for-length *z*-score (WLZ) for enrolment to malnutrition treatment programmes but acknowledge a weak evidence-base. This review aims to inform future guidelines by examining which anthropometric criteria best identify infants <6 m at high risk of mortality/morbidity.

**Methods:**

We searched Medline, EMBASE, CINAHL, Global Health, Cochrane Library and POPLINE for studies conducted in low- and middle-income countries and published between 1990 and October 2020. We included studies reporting anthropometric assessment of nutritional status in infants <6 m and assessed the association with subsequent morbidity or mortality.

**Results:**

A total of 19 studies were included in the final review, covering 20 countries, predominantly in sub-Saharan Africa. WLZ had poor reliability and poor prognostic ability to identify infants at risk of death. Mid-upper arm circumference (MUAC) and weight-for-age *z*-score (WAZ) were better at identifying infants at risk of mortality/morbidity. MUAC-for-age *z*-score did not perform better than using a single MUAC cut-off. Suggested MUAC cut-offs for this age group varied by context, ranging from 10.5 to 11.5 cm. The assessment for reliability showed that length was difficult to measure, making WLZ the least reliable indicator overall.

**Conclusion:**

Evidence from our review suggests that a change in current practice is necessary. To better identify small and nutritionally at-risk infants <6 m WAZ and/or MUAC rather than WLZ should be used. Future research should explore possible benefits for programme coverage, impact and cost-effectiveness. Research should also examine if context-specific MUAC thresholds are needed.

## Introduction

Approximately 8.5 million infants less than 6 months old (<6 m) globally are wasted.^1^ These infants are especially vulnerable to the short- and long-term health risks associated with anthropometric deficits. They face significantly higher risk of morbidity and mortality than older malnourished children,^2^ and in the long-term, potentially impaired physical and cognitive development, including greater susceptibility to cardiovascular and metabolic non-communicable diseases.^3-7^

Current wasting interventions focus on children 6 to 59 months with infants <6 m overlooked or neglected in many treatment programmes.^8^ There is growing appreciation that apparent wasting in this age group needs to be taken more seriously, understood and better addressed.^9^ Whilst supporting exclusive breastfeeding is the main route to appropriate nutrition in this age group, only 37% of infants <6 m are exclusively breastfed in low- and middle-income countries.^10^ Even then, exclusive breastfeeding may not fully protect an infant against undernutrition since many other factors can contribute to inadequate weight gain. Cultural and socioeconomic influences, maternal nutritional deficits, maternal mental health problems, breastmilk composition (particularly oligosaccharides and bioactive proteins), acute and chronic infections, enteropathy, birth defects and prenatal nutrition can all negatively influence nutrition and growth in infants.^11,12^ The current COVID-19 pandemic poses additional problems in these areas. National and international food and health systems that were already fragile are now under significantly greater stress, with associated practical difficulties finding, assessing and supporting at-risk mothers and infants.^13^

Current World Health Organization (WHO)^14^ guidelines on the ‘Management of Severe Acute Malnutrition in infants and children’, published in 2013, include a chapter focussing on the unique needs of infants <6 m. The guidelines recommend using low weight-for-length *z*-scores (WLZ) to identify small and nutritionally at-risk infants <6 m for admission to treatment programmes. However, this guidance was not based on direct evidence review but on conventions used among older children. In a 2015 systematic prioritisation of research questions to improve the management of malnourished infants <6 m flagged ‘defining wasting in this age group’ as the top priority question out of 60 listed.^15^

Since the publication of the 2013 WHO guidelines and the 2015 prioritisation review, there has been growing appreciation that anthropometric case definitions of malnutrition should focus on those best able to predict clinically significant events – notably mortality and morbidity.^16^ A small but growing number of studies have explored the prognostic value of different anthropometric criteria, as well as their applicability and feasibility in front-line nutrition programmes.^17,18^ National and international guidelines are currently being updated to reflect those new perspectives and priorities. Evidence to inform these updates is urgently needed. Our systematic review therefore aims to synthesise latest evidence on which anthropometric criteria best identify infants at-risk of morbidity and mortality.

## Methods

Our systematic review protocol was pre-registered with PROSPERO (#CRD42019141047). The London School of Hygiene and Tropical Medicine Research Governance & Integrity Office also reviewed the methodology but since we only use publicly available sources decided that no formal ethical approval was required.

We based our search strategy on a previous literature review exploring admission and discharge criteria for malnourished infants <6 months.^19^ We considered anthropometric measures as risk factors for morbidity and mortality, and also reviewed quality, reliability and validity of measurements. Studies that only compared how 1 anthropometric criterion predicted another anthropometric criterion were excluded since this design has no gold standard measure. Table 1 summarises our PICOSS framework:

**Table 1.** Screening strategy framework.

Framework	Search specifics
Population	Infants aged <6 m
Intervention	Studies that assessed short term nutritional status in infants <6 m using anthropometric measurements
Comparator	Not applicable, since we considered all study designs
Outcome	Studies reporting on associated morbidity and mortality, or sensitivity, specificity or predictive value of anthropometric deficit
Setting	All low- and middle-income countries
Study design	All eligible

The following databases were searched: Medline, EMBASE, CINAHL, Global Health, Cochrane Library and POPLINE. The search was filtered to include only articles in English, research in humans and publications since 1990. Additional studies were found through reviewing the reference lists of the identified publications. Full list of search terms can be found in Supplemental Annex 1. The last search was run on 15th October 2020.

All initial results were screened by the first author based on their title and abstract; any studies that could not be excluded were then evaluated for inclusion based on the full text. Data were extracted from eligible studies using a standardised form to capture information regarding participants and setting; indicators measured; outcomes reported; and results.

Quality assessment was done based on the ‘Methods for the development of NICE (National Institute for Health and Care Excellence) public health guidance’.^20^ Due to the small number and heterogeneity of studies, the analyses are presented as a narrative synthesis. The presentation of the evidence follows a comparison of the different indicators based on the conceptual framework by Myatt et al,^21^ focussing on the prognostic properties of each indicator and their quality.

## Results

**Figure 1.**
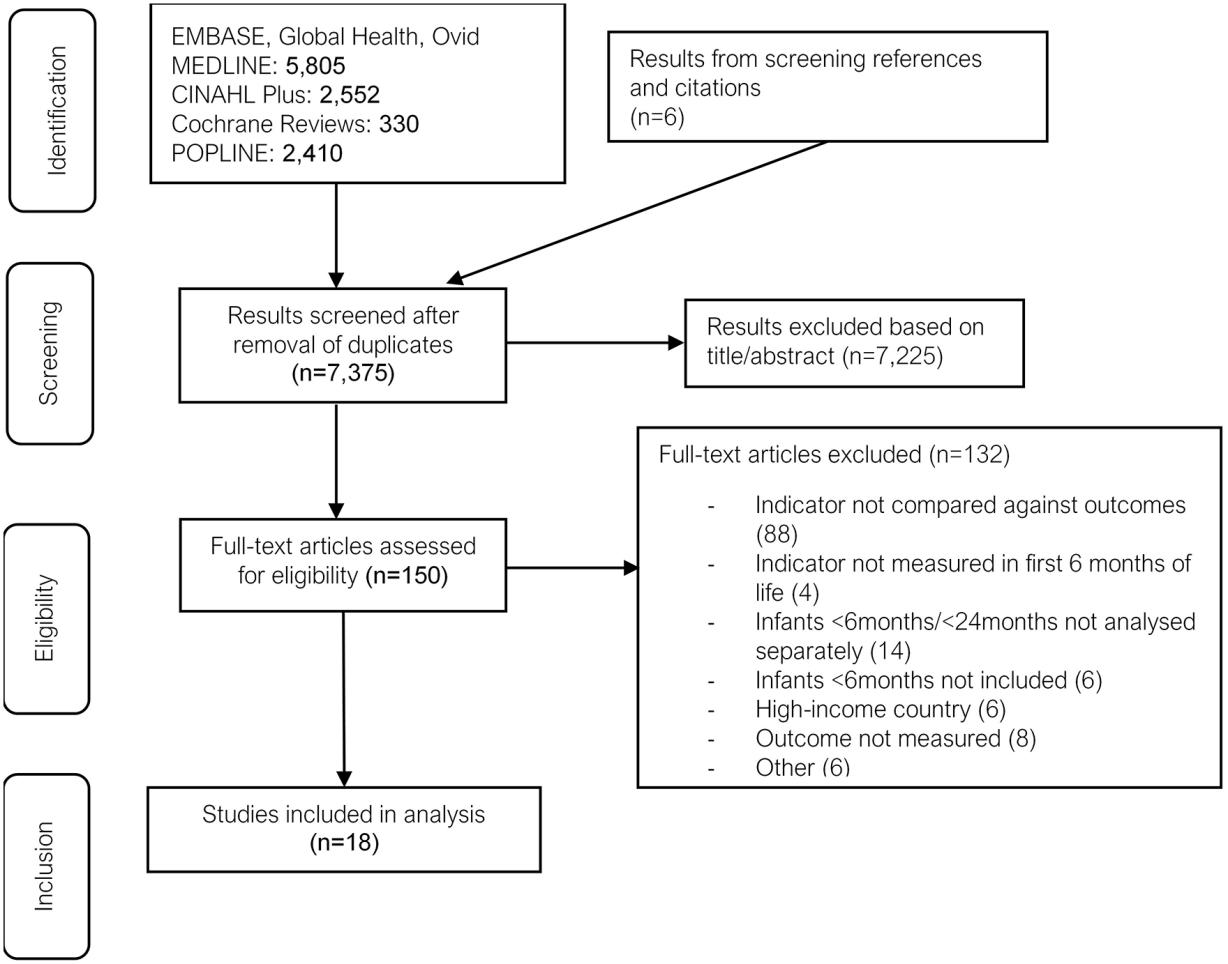
PRISMA flow diagram of screening process.

After removing duplicates, 7375 titles and abstracts were identified; 150 full texts were reviewed; and 18 studies were identified for final inclusion in the analysis (see Figure 1 for flow diagram of screening process). The studies included represent 20 countries and are either prospective cohorts relating to outcomes or cross-sectional study designs relating to reliability or applicability (Table 2). Almost all the studies (14/18) took place in sub-Saharan Africa; 2/18 are from Asian countries; and 2/18 are set across multiple countries. The number of participants in the studies varied between 250 and 48 492, although the latter did not report the number of infants <6 m separately. A total of 12 assessed predictive value of anthropometric indicators; 5 studies assessed quality of anthropometric indicators. Out of the 12 studies analysing anthropometric indicators against subsequent morbidity or mortality, 9 studies included MUAC, 7 studies included weight-for-age *z*-score (WAZ) and 9 included weight-for-length *z*-score (WLZ). Three studies compared all 3 indicators. Other criteria investigated were MUAC-for-age *z*-score (MUAC-FA) (n = 4), length-for-age *z*-score (LAZ) (n = 7), BMI-for-age *z*-score (BMI-FA) (n = 1) and weight-velocity for age (WV-FA) (n = 1). Of the 7 studies analysing infants <6 m separately, 2 were set in a hospital,^22,23^ and 5 in the community.^24-28^ Of the 5 studies analysing infants <6 m together with infants up to 24 months of age, 1 was set in a hospital^29^ and 4 in the community.^30-33^

**Table 2.** Summary of included studies.

Author	Country	n	Population	Anthropometry	Key results and conclusions
*Studies analysing predictive value in infants* <*6 mo only*					
Mathenge et al^23^	Kenya	3432	2-6 m, inpatients	MUAC	MUAC < 11 cm occurred in 19% of infants and was associated with case fatality of 23% compared to case fatality of 5% for MUAC > 11 cm (*P* < .001, relative risk 6.64 [95%CI 4.08-10.8]).
Mwangome et al^25^	Burkina Faso	1103	Birth cohort	WLZ, WAZ, MUAC	At age 2 mo, MUAC < 11.5 cm, WAZ < −3 and LAZ < −3 all predicted death. WLZ was not associated with mortality at any threshold.
Mwangome et al^22^	Kenya	2882	<6 m, inpatients	WLZ, WAZ, MUAC, MUAC-FA	MUAC and WAZ predicted inpatient and post-hospital discharge mortality better than WLZ (*P* < .0001). MUAC < 11.0 cm performed similarly to MUAC-FA (*P* > .05) and better than WLZ < −3 for both inpatient and post-discharge mortality (*P* < .001)
Mwangome et al^24^	The Gambia	2876	6-14 wk, in the community	WLZ, MUAC	The areas under the ROC curve for death in infancy were 0.55 (95% CI: 0.46-0.64) for WFLz and 0.64 (95% CI: 0.55-0.73) for MUAC. MUAC < 11.0 cm predicted mortality better than WLZ < −3.
Rasmussen et al^28^	Guinea-Bissau	11 614	<3 y, in the community	MUAC, MUAC-FA	No difference in sensitivity and predictive value for the lowest 5% of MUAC and MUAC-FA *z*-scores, for mortality within 90 d of follow-up among infants aged 3-35 mo. No MUAC-FA reference available for infants 0-2 mo.
Vella et al^27^	Uganda	4320	<5 y, in the community	WLZ, LAZ, WAZ, MUAC	MUAC was the most sensitive predictor of mortality at 12-mo follow-up, followed by WAZ, LAZ, then WLZ. MUAC < 12.5, 11.5 and 10.5 cm predicted 11%, 19% and 37% of deaths respectively
Vesel et al^26^	Ghana, India, Peru	8787	<6 m, in the community	WLZ, WAZ	WAZ < −3 at the first immunisation visit had the highest sensitivity (70.2%) and specificity (85.8%) for predicting mortality in India. Indicators could not be assessed in Ghana or Peru due to insufficient number of outcome events.
*Studies analysing predictive value in infants* <* 6 mo together with older infants and children*					
Gernaat et al^29^	Zambia	299	0-59 mo, with SAM	MUAC, LAZ	MUAC < 10.4 cm (OR 0.33) and LAZ < −3 (OR 0.57) predicted mortality in infants 0-18 mo of age
O’Neill et al^30^	DRC	2402	<24 m, in the community	WLZ, MUAC-FA, WV-FA, BMI-FA	All indicators predicted mortality (LAZ < −3, BMI-FA < −2, MUAC-FA < −3, WV-FA < −2). The highest hazard ratio point estimate was in children with BMI-FA, <−3 *z*-scores (HR = 9.11).
Tonglet^31^	DRC	842	<24 m, in the community	WAZ, LAZ, WLZ, MUAC-FA, WV-FA	No anthropometry performed well at predicting general morbidity but all anthropometry <25th centile increased risk of diarrhoea. Non-anthropometric criteria (reported growth by caregiver; diet; recent illness) were more useful.
Vella et al^32^	Uganda	1178	0-59 m, in the community	WLZ, WAZ, LAZ	After 12 mo of follow-up, WAZ was most sensitive predictor of mortality at specificities >88%; WLZ was more sensitive at lower specificities. LAZ was not associated with mortality.
Van den Broeck et al^33^	DRC	5167	0-59 m, in the community	WLZ, WAZ, LAZ, MUAC-FA	All indicators predicted mortality. Short-term mortality (within 3 mo) was significantly associated with WAZ < −3, LAZ < −4, WLZ < −1, MUAC-FA < −3 and kwashiorkor. The relative risks were extremely high for WAZ < −4 and MUAC-FA < −4
*Studies analysing MUAC thresholds as a predictor of morbidity or mortality*					
Gupta et al^34^	Nepal	48 492	<3 y, in the community	MUAC	MUAC < 11.5 cm predicted mortality in 1 ethic group but not another (Pahadis HR = 1.12; 95% CI 0.72-1.73, Madeshis HR = 1.76; 95% CI 1.35-2.28).
*Studies analysing the quality of anthropometric indicators*					
Ayele et al^35^	Ethiopia	606	0-5 y, in the community	MUAC, weight, length	Reproducibility in community settings was higher for weight and length than MUAC. Intra-examiner relative TEM was 0.35% for height, 0.39% for weight and 1.27% for MUAC. *z*-score transformed data were not evaluated.
Jamaiyah et al^36^	Malaysia	130	0-2 y	Weight, length	Weight measures were more reliable and valid than length measures in a hospital outpatient clinic. The relative TEMs for inter and intra-examiners for weight were 0.8% and 1.1% respectively; length were 2.1% and 1.9%. *z*-score transformed measures were not evaluated.
Mwangome et al^37^	Kenya	924	<6 m, in the community	WLZ, MUAC	MUAC was more reliable than WLZ, measured by community health workers. Intra-class correlations were 0.96 (95% CI 0.95-0.96) for MUAC and 0.71 (95% CI 0.68-0.74) for WLZ
Onis^38^	Multi-country*	1800	0-24 m	MUAC, length	Under research conditions, MUAC was slightly more reliable than length; the coefficient of reliability was above 95% for all measurements. *z*-score transformed measures were not evaluated.
Ezeofor et al^39^	Nigeria	411	0-6 m, inpatients	WAZ, MUAC, WLZ	Low WAZ was the most discriminate predictor of ‘weight faltering’ (sensitivity 69%, ppv 86%, likelihood ratio 5.5; area under ROC 0.90) followed by MUAC (73%, 73%, 4.9; 0.86), while WLZ performed least well (49%, 67%, 2.9; 0.84).

Abbreviations: BMI-FA, BMI for age; DRC, Democratic Republic of Congo; LAZ, length for age *z*-score; MUAC, mid-upper arm circumference; MUAC-FA, MUAC-for-age *z*-score; TEM, technical error of measurement; WAZ, weight for age *z*-score; WLZ, weight for length *z*-score; WV-FA, weight velocity for age (over a 3-month period).

* Brazil, Ghana, India, Norway, Oman, USA.

Overall, the studies showed a low to moderate risk of bias across different domains (see detailed scores in Supplemental Annex Table 1). The highest risk of bias was found in the domains of *Study Attrition* and *Study Confounding*, due to a lack of detailed reporting about how the authors dealt with loss to follow-up and confounding variables.

Our review found that WLZ performed poorly when assessed for its prognostic ability and reliability. Six studies^22,24-27,32^ found no or very delayed association between WLZ and mortality. The studies all had a relatively long follow-up period (4.5-10.5 months) but none collected details about the causes of death.

MUAC and WAZ identified infants at risk of mortality and morbidity better than WLZ.^22,24,26,27^ MUAC-FA *z*-score did not perform better than using a single MUAC cut-off.^28^ The most appropriate cut-off for this age group varied by context, ranging from 10.5 to 11.5 cm. In Kenya, Mwangome et al^22^ found a threshold of <11.0 cm to be statistically optimal for identifying inpatient mortality risk and < 11.5 cm for identifying those at risk of post-discharge mortality . In Burkina Faso <11.5 cm identified infants at risk of mortality reliably, but also captured a large proportion of infants overall (17%).^25^ A cut-off of <11.0 cm identified 6.8% of infants, which may be more reasonable depending on the available resources. However, in The Gambia, <11.0 cm captured 14% of the population.^24^ In a study in Nepal, MUAC <11.5 cm predicted mortality in 1 ethnic group (Madeshis, HR = 1.76; 95% CI 1.35-2.28) but not another (Pahadis, HR = 1.12; 95% CI 0.72-1.73).^34^

In assessments of measurement reliability, length was generally more difficult to measure accurately, meaning WLZ is often the least reliable of the indicators.^36-40^ Ezeofor et al^39^ reported WLZ had the lowest sensitivity under 3 months and overall detected only half of all cases of ‘undernutrition’ (defined as weight faltering or sum of skinfolds <10 mm), while WAZ had the highest positive predictive value at all ages (0-6 months). Two studies specifically note the issue that WLZ cannot be calculated on infants less than 45 cm^22,25^; this only affected 0.2% of infants in a community-based study but affected 15% in a hospital-based study. Ayele et al^35^ reported measurement errors for weight, length and MUAC by members of a rural community in children 0 to 69 months in Ethiopia and found slightly higher errors for smaller children than for larger ones. The relative Technical Error of Measurement (%TEM) for MUAC (1.48 [95% CI 1.26-1.69]) was higher than for height (0.80 [0.68-0.92]) and weight (0.78 [0.66-0.91]. In contrast, results from the *WHO Multicentre Growth Reference Study*, conducted under optimal research circumstances, found MUAC to have slightly lower TEM than length in children <24 months.^38^ Mwangome et al^37^ found length to be less reliable than weight and MUAC in infants <6 m, and identified that errors in length and weight measurement were magnified in the WLZ calculation, making WLZ the least reliable indicator overall. Jamaiyah et al^36^ also found length (%TEM 2.08) in children <2 years to be slightly less reliable than weight (%TEM 0.79). No studies formally assessed acceptability, cost or simplicity of measurements which are additional factors to consider, as per Myatt et al’s^21^ framework.

## Discussion

Our review found a small number of high-quality studies assessing the relationship between anthropometric indicators of nutrition status and subsequent risk of mortality in infants <6 m. Most found MUAC and/or WAZ to be the best predictors of short-term mortality, as well as having lower measurement errors than length-based measurements. There was little benefit of using a MUAC-FA measure over a fixed threshold.

The current standard indicator for identifying at-risk infants <6 m is WLZ. We found 8 studies that investigated the predictive ability of WLZ for mortality and in all the studies WLZ was either not associated with subsequent mortality, performed equally well as the other indicators or showed the worst performance. Additionally, several studies investigated the reliability of measuring length in infants and all found it to be a consistently less reliable measure than weight. This could be due to several factors including: the physiological tendency of young infants to flex their knees and hips, making proper flat positioning on a measuring board challenging; healthcare workers’ relative unfamiliarity handling young infants; the fact that infants are active and difficult to hold still. *z*-score calculations of WAZ and WLZ add opportunity for additional errors, and magnifies existing errors in weight or length, especially for WLZ which combines the 2 measures. An additional problem is that WLZ cannot be computed for infants shorter than 45 cm – hence some missing values for this indicator. Whilst the WHO reference shows that by 4 weeks of age, 99% of infants are longer than 45 cm, those that are not are among the most vulnerable ones.^41,42^ Based on these findings, it is difficult to justify continuing to recommend WLZ as the standard indicator for nutritional vulnerability in infants <6 m.

For assessing nutritional risk in older children (6-59 months), the common alternatives to WLZ are MUAC and occasionally WAZ. This review found that WAZ was a predictor of mortality in 6/6 studies. Weight is routinely assessed in infants in growth monitoring programmes, so WAZ also has the advantage of familiarity and that scales are widely available and health workers are trained to use them. Age used to be perceived as problematic to ascertain accurately but increasing numbers of infants worldwide have date of birth recorded and even where not available it is easier to calculate than in older children since shorter recall periods are needed.^21,40^ WAZ has also been previously dismissed as a useful indicator as it is ineffective at differentiating between deficits in height versus deficits in weight, however recent studies in older children (6-59 months) have highlighted the higher risk of death in children with multiple nutritional deficits and WAZ is better at identifying these children than WLZ.^17,43^ Use of WAZ rather than WLZ would also improve practicalities of assessing risk at a community level, although MUAC is the most useful measure in this regard.

For MUAC, studies of its predictive value, precision, accuracy and independence of age were found. All studies of infants <6 m showed an association between MUAC and mortality. The 3 studies that analysed whether MUAC was independent of age found its performance was either not influenced by age or performed equally well when compared to MUAC-FA. The cut-off threshold used has a large impact on specificity and sensitivity for identifying at-risk infants and optimal cut-off values seemed to vary by context. Statistically optimal cut-off values ranged from 11.5 to 10.5 cm, each with consequences to be considered for types of intervention and trade-offs between sensitivity and burden on treatment programmes.^24,25,44^ MUAC is the cheapest indicator to measure and requires only minimal training.^18,45^ It is also well accepted by mothers as infants do not need to be undressed and can remain in the arms of the caretaker.^46^ It can also be accurately conducted by mothers themselves at home.^47,48^ These benefits strongly suggest that MUAC should be included in the nutritional assessment of infants <6 m in order to improve efficiency, coverage and effectiveness of identification; it is already recommended by the recently updated *MAMI Care Pathway ( Management of small & nutritionally At-risk Mothers and Infants)*.^49^ Future research is needed to represent a wider range of contexts and identify context-specific MUAC thresholds. Ideally, this research should compare indicators against clinical outcomes rather than against each other.

This is the first systematic review examining anthropometric criteria specifically for infants <6 m, and is not without limitations. Significant heterogeneity in results, including which anthropometric indicator was analysed, at what age, how frequently and with what case definition, limits the comparability of findings. Four of the studies were also relatively old, having taken place more than 20 years ago. This may affect their generalisability to current populations. Another limit to the generalisability, is that some of the highest burden countries for wasting are not represented, such as those in the African Sahel region, and only 1 study for South America and 1 study from South-East Asia were found.

Future research should address these limitations and examine the different anthropometric indicators in a wider range of current settings. Though it is plausible that simpler indicators, notably MUAC might facilitate improved programme coverage and impact and contribute to greater cost-effectiveness of treatments, this needs to be formally explored. Can MUAC be used as a stand-alone indicator or is combination with WAZ better to identify higher risk infants? Towards this, more data is needed to determine MUAC cut-off recommendations. One cut-off for the whole 0 to 6 m age range is unlikely but perhaps there could be: 1 cut-off for early infancy; another for 6 weeks (age of first vaccination) to 6 months. Future research should also examine how proposed new indicators might improve links with other programmes: WAZ with growth monitoring programmes; MUAC embedded into vaccination and community programmes for example. Finally, it is important to document outcomes for infants who might not be identified using the new criteria, for example, the small numbers with low WLZ but normal MUAC and normal WAZ.

## Conclusion

While further research remains necessary, we found good quality evidence to underpin changes to current policy and practice relating to small and nutritionally at-risk infants <6 m. To better identify infants at high risk of mortality and morbidity, WAZ and/or MUAC should be used in preference to current WLZ criteria. MUAC-for age does not add value but more data is needed to determine what MUAC cutoffs should be used. Future research is needed to explore the performance of the various indicators in a wider range of contexts and to document their impact on programme coverage, impact and cost-effectiveness.

## Supplemental Material

sj-docx-1-pdi-10.1177_11795565211049904 – Supplemental material for Anthropometric Criteria for Identifying Infants Under 6 Months of Age at Risk of Morbidity and Mortality: A Systematic Review Click here for additional data file. Supplemental material, sj-docx-1-pdi-10.1177_11795565211049904 for Anthropometric Criteria for Identifying Infants Under 6 Months of Age at Risk of Morbidity and Mortality: A Systematic Review by Christoph Hoehn, Natasha Lelijveld, Martha Mwangome, James A Berkley, Marie McGrath and Marko Kerac in Clinical Medicine Insights: Pediatrics
