# Supplementary material for: Anthropometric Criteria for Identifying Infants Under 6 Months of Age at Risk of Morbidity and Mortality: A Systematic Review
Source: Clin Med Insights Pediatr. 2021 Oct 21;15:11795565211049904. doi: 10.1177/11795565211049904 (PMC8543668; doi:10.1177/11795565211049904)
Supplement: sj-docx-1-pdi-10.1177_11795565211049904 – Supplemental material for Anthropometric Criteria for Identifying Infants Under 6 Months of Age at Risk of Morbidity and Mortality: A Systematic Review [file sj-docx-1-pdi-10.1177_11795565211049904.docx]

**Supplementary material**

**Anthropometric criteria for identifying infants under 6 months of age at risk of morbidity and mortality: a systematic review**

**Annex 1: Full search terms**

*General search concepts*

| **Concept 1: Population** | |
| --- | --- |
|  | Infant, infants, |
|  |  |
| **Concept 2: Intervention** | |
|  | Growth Disorders, Infant Nutrition Disorders, Anthropometry, Thinness, MUAC, WAZ, WLZ |
|  |  |
| **Concept 3: Outcome** | |
|  | Hospitalization, Infant Mortality, Infant Death, Morbidity, Sensitivity and specificity, Predictive value of tests, |
|  |  |
| **Concept 4: Setting** | |
|  | All low- and middle-income countries |

**CINAHL Search:**

((MH "Infant") OR infant OR infants OR infancy OR babies OR baby OR neonate OR neonates OR newborn* OR new-born*)

*AND*

((MH "Infant, Hospitalized") OR (MH "Infant Mortality") OR (MH "Sensitivity and Specificity") OR (MH "Reliability and Validity") OR (MH "Predictive Validity") OR (MH "Predictive Value of Tests") OR death OR "case fatality" OR hospitali?ation* OR mortalit* OR morbidit* OR sensitivity OR specificity OR reliab* OR valid*)

*AND*

((MH "Anthropometry") OR (MH "Thinness") OR anthropometry OR linear growth OR "growth disorder*" OR "infant growth" OR wasting OR wasted OR weight-for-age OR WFL OR WLZ OR MUAC OR mid-upper-arm-circumference OR WFA OR WAZ OR underweight OR thinness OR weight-for-length)

**Cochrane Search:**

(infant OR infants OR infancy OR babies OR baby OR neonate OR neonates OR newborn* OR new-born*)

*AND*

(mortalit* OR morbidit* OR death OR "case fatality" OR hospitali?ation* OR sensitivity OR specificity OR reliab* OR valid* OR "Predictive Valid*" OR "Predictive Value*")

*AND*

(anthropometry OR linear growth OR "growth disorder*" OR "infant growth" OR wasting OR wasted OR weight-for-age OR WFL OR WLZ OR MUAC OR mid-upper-arm-circumference OR WFA OR WAZ OR underweight OR thinness OR weight-for-length)

**POPLINE Search (only until July 2019 as the database was decommissioned after this point):**

(infant OR infants OR infancy OR babies OR baby OR neonate OR neonates OR newborn* OR new-born*)

*AND*

(mortality OR morbidity OR death OR "case fatality" OR hospitalization OR hospitalizations OR mortalities OR morbidities OR hospitalisation OR hospitalisations)

*AND*

(anthropometry OR linear growth OR "growth disorder*" OR "infant growth" OR wasting OR wasted OR weight-for-age OR "weight for age" OR WFL OR WLZ OR MUAC OR "mid* upper arm circumference" OR mid-upper-arm-circumference OR WFA OR WAZ OR underweight OR thinness OR "weight for length" OR weight-for-length)

**Annex Table 1: Risk of bias assessment**

| Risk of bias assessment | | | | | | |  |
| --- | --- | --- | --- | --- | --- | --- | --- |
| Author (Year) | Quality* | | | | | |  |
|  | P | A | F | O | C | S | |
| *Studies analysing infants < 6 months of age* | | | | | | |  |
| Mathenge (2009) |  |  |  |  |  |  | |
| Mwangome (2019) |  |  |  |  |  |  | |
| Mwangome (2017) |  |  |  |  |  |  | |
| Mwangome (2012) |  |  |  |  |  |  | |
| Rasmussen (2012) |  |  |  |  |  |  | |
| Vella (1994) |  |  |  |  |  |  | |
| Vesel (2010) |  |  |  |  |  |  | |
| *Studies analysing infants up to 24 months of age* | | | | | | |  |
| Gernaat (1998) |  |  |  |  |  |  | |
| O’Neill (2012) |  |  |  |  |  |  | |
| Tonglet (1999) |  |  |  |  |  |  | |
| Vella (1992) |  |  |  |  |  |  | |
| Van den Broeck (1993) |  |  |  |  |  |  | |
| *Studies analysing MUAC thresholds* | | | | | | |  |
| Gupta (2018) |  |  |  |  |  |  | |
| *Studies analysing the quality of the indicators* | | | | | | |  |
| Ayele (2012) |  | x |  |  |  |  | |
| Jamaiyah (2010) |  | x |  |  |  |  | |
| Mwangome (2012) |  | x |  |  |  |  | |
| Onis (2006) |  | x |  |  |  |  | |
| Ezeofor (2020) |  | x |  |  |  |  | |

*Red=high risk of bias, yellow=moderate risk of bias, green= low risk of bias

P = Study Participation

A = Study Attrition

F = Prognostic Factor Measurement

O = Outcome Measurement

C = Study Confounding

S = Statistical Analysis and Reporting

x = not applicable
